# Design and Application of Uniaxially Sensitive Stress Sensor

**DOI:** 10.3390/mi16010094

**Published:** 2025-01-15

**Authors:** Kaituo Wu, Zixun Xiang, Xinbo Lu, Yichao Yan, Chunyang Wu, Tao Wang, Wanli Zhang

**Affiliations:** State Key Laboratory of Electronic Thin Films and Integrated Devices, University of Electronic Science and Technology of China, Chengdu 611731, China

**Keywords:** stress sensor, uniaxial sensitive, Wheatstone bridge

## Abstract

Current stress sensors for microsystems face integration challenges and complex signal decoding. This paper proposes a real-time uniaxially sensitive stress sensor. It is obtained by simple combinations of bar resistors using their sensitivity differences in different axes. With the aid of a Wheatstone bridge, the sensor can measure the uniaxial stress magnitude by simple calibration of the stress against the output voltage and detect the bidirectional stress magnitude and direction in a micro-zone by simple rotation. The theoretical sensitivity obtained from simulation is 0.087 mV/V·MPa when the X-bridge is stressed in the X-direction under 1 V of excitation, and the test sensitivity of the X-bridge prepared in this paper is 0.1 mV/V·MPa. The design is structurally and procedurally simple, exhibits better temperature stability, and reduces interface requirements, making it suitable for the health monitoring of multi-chip microsystem chips.

## 1. Introduction

Microsystems technology integrates sensors, controllers, actuators, and other multi-functions through system architecture and software algorithms, and adopts micro/nano- manufacturing and micro-integration processes to realize the micro/nanoscale system structure, which is a kind of cutting-edge science and technology integrating microelectronics, micro-electromechanics, and micro-optics technologies and has the advantages of high integration, micro-miniaturization, low power consumption, and multi-functionality.

**Figure 1:**
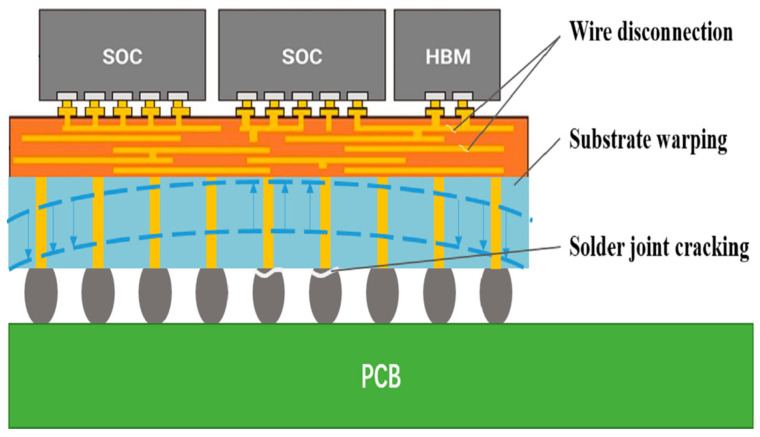
Typical failure examples (Wire disconnection, Substrate warping, Solder joint cracking).

As the integration of microsystems increases, factors such as packaging and heat generation lead to a significant increase in the internal stresses of the system, with the mismatch of the coefficient of thermal expansion and packaging stresses being particularly prominent. Microsystems are composed of a variety of materials (e.g., silicon, metals, and ceramics) with different coefficients of thermal expansion, which produce different degrees of expansion or contraction when the temperature changes, thus triggering internal thermal stresses. At the same time, the mechanical constraints between the packaging material and the chip during the chip packaging process also introduce stresses. These stresses not only lead to material fatigue and damage, increasing the risk of desoldering point failure, but also lead to device performance parameter drift during temperature changes or prolonged operation, affecting the stability and consistency of the chip. Figure 1 gives some examples of typical failures that lead to device failure including cracked solder joints, warped substrates, broken leads, etc. Therefore, in order to ensure the normal operation of the chip within the microsystem, it is particularly important to monitor the stress parameters of the key components of the chip [1,2,3,4,5]. Currently, the stress sensors applied to microsystems are difficult to integrate with the system, and the stress sensors need to decode the output signal, which is not easy to apply [6,7,8,9,10,11,12,13,14].

Methods used to measure micro-area stress are mainly metal thin-film stress sensors and semiconductor thin-film stress sensors [15]. Among them, metal thin-film stress sensors usually use strain-volume-induced changes in metal or alloy resistance to measure stress, have the advantages of good stability, and are applicable to a wide range of temperatures, etc., but their sensitivity coefficient is low, and in order to obtain a larger output resistance, there needs to be a large number of winding metal resistors, resulting in a low degree of integration. Semiconductor thin-film stress sensors use strain-induced resistivity changes to measure stress [16,17,18], have high-sensitivity coefficients, and are easy to integrate and mass produce, but their preparation process is more complex, and signal processing is inconvenient. In addition, the traditional semiconductor thin-film stress sensors are mainly patch-type, using adhesive and other methods to combine with the measured parts, through stress transfer measurement [19,20,21,22,23]. There are shortcomings such as stress transfer attenuation, lead complexity, introduction of load error, and difficulty in integration, which makes it difficult to apply to online stress detection inside multi-chip packaged microsystems [24,25,26,27,28].

Multi-chip-encapsulated microsystems are mainly integrated through the substrate/adapter plate to achieve integrated packaging, and thin-film stress sensors integrated in the microsystem packaging substrate, in the packaging process to complete the sensor chip batch embedding, can achieve the key microsystem parameters of the in situ measurement. At present, there are many problems with the above integration and testing methods, the most important of which is the limited number of embedded sensor input/output (I/O) interfaces in the microsystem. Specifically, there is stress in the microsystem in multiple directions; to measure the stress in each direction within the surface, the piezoresistive coefficients in each direction are calculated by the rosette resistor strip, and in the calibrated measurement method, at least four I/O interfaces are required and at least eight interfaces are required by the Kelvin measurement method, which makes the sensors in the microsystems lead out to cause a high overhead of the I/O interfaces, especially to realize the state of the stresses within the microsystems. When inversion is required, arrayed embedded sensors are needed, and the number of I/O interface overheads is unacceptable for the microsystem. In addition, the accuracy of the sensors fabricated by this method has a large effect with temperature, making it difficult to use in high-temperature environments [29,30,31,32,33,34].

To address the above difficulties in existing applications, this paper provides a simulation design of a single-axis-sensitive integrated stress sensor and its preparation method, and experimentally verifies the feasibility of the design. This sensor does not need to solve the output signal to solve for the stress component, and the measurement of the unidirectional stress magnitude can be realized by the voltage output through a simple calibration of the stress versus the output voltage. In addition, the magnitude and direction of bi-directional stress can be detected in the micro-region by a simple combination of rotations. The method is simple in structure and preparation process, and it not only has better temperature stability but also reduces the need of an I/O interface, which can be popularized and applied to the health monitoring of multi-chip microsystem chips.

## 2. Materials and Methods

This paper uses the COMSOL Multiphysics (Version 5.6) finite element simulation tool to guide the design of the sensor. In order to ensure simulation accuracy and optimization performance, the simulation structure and model are appropriately simplified to improve the mesh quality, accelerate the convergence, and reduce the computing time. The modeling steps are roughly summarized as follows. Firstly, a solid block with a square top view is constructed, and the sensor structure to be computationally verified is subsequently stretched out on its top surface. In the second step, the material properties of all the solids are set to p-Silicon (single-crystal, lightly doped) from the material library, and some basic parameter values are shown in Table 1. The third step is to set the boundary conditions; except for the two sides under force, the boundary conditions of the other two sides and the bottom side are set to “Roller”, which constrains the normal displacement (or motion) of the boundary and only allows the tangential direction to have displacement. The fixed constraints are on the origin of the chip coordinates. This setting reduces unnecessary constraints. In the fourth step, in the “Electric Currents” setting, the sensor part is set to piezoresistive material and the doping region is uniformly p-type. The terminals and their voltage or current values are set as required. Finally, the piezoresistive effect multiphysics fields are applied to the model.

### 2.1. Rectangular Resistor

**Figure 2:**
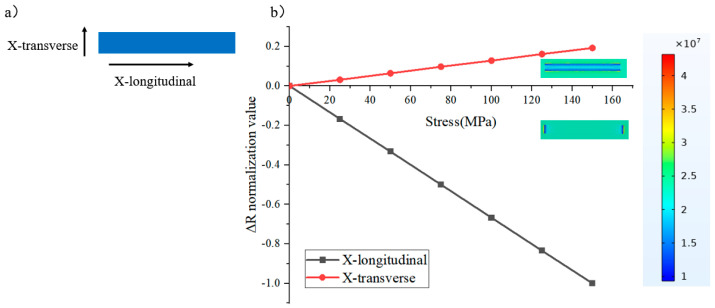
(**a**) X-bar and its (**b**) sensitivity in different directions.

First, a rectangular resistor with a piezoresistive effect is shown in Figure 2a (X-bar). When a force of equal magnitude is applied to it in the X-longitudinal and X-transverse directions, the resistance changes as shown in Figure 2b. As can be seen from the COMSOL Multiphysics simulation results, the sensitivity of the resistor to the X-longitudinal stress is significantly greater than its sensitivity to the X-transverse stress, and there is a difference of more than a factor of 5 between them. By rotating this resistor by 90°, it is easy to obtain a sensor that is sensitive to the Y-longitudinal directions (Y-bar).

**Figure 3:**
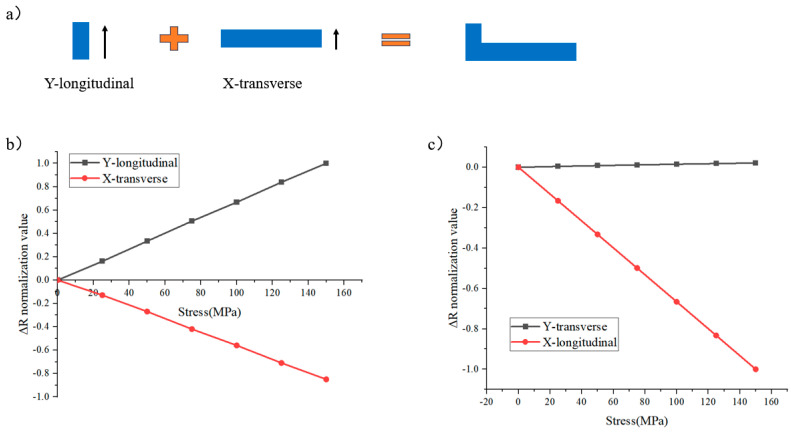
(**a**) The combination of Y-bar and X-bar with their (**b**) sensitivity offset; (**c**) the sensitivity of the X-sensor.

On this basis, by adding a small section of the Y-bar to the X-bar (Figure 3a), the longitudinal sensitivity of the Y-bar counteracts the transverse sensitivity of the X-bar to achieve a sensor sensitive only to the x-direction, and by rotating it by 90°, a sensor sensitive only to the y-direction can be obtained, which is named the X-sensor and Y-sensor.

Figure 3c shows the sensitivity of the X-sensor; the sensitivity in the Y-transverse direction is close to zero and the sensitivity in the X-longitudinal direction is much greater than that in the Y-transverse direction, so it can be considered that this L-shaped sensor is capable of uniaxial stress sensing.

**Figure 4:**
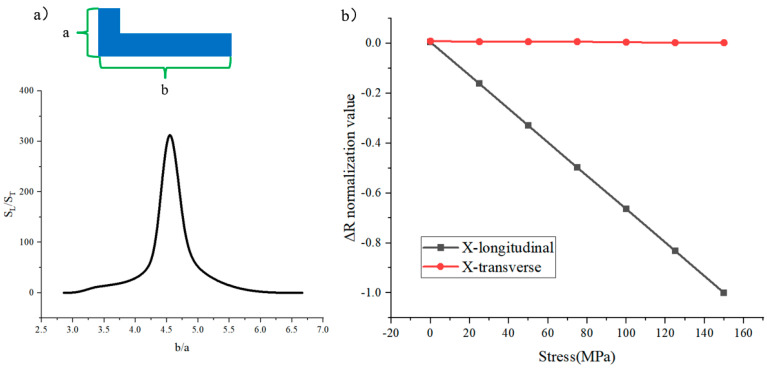
(**a**) Optimization of the sensor’s length–width ratio; (**b**) The sensitivity in both directions at b/a equal to 4.5.

To maximize the sensitivity of the L-sensor, a simulation study on the length–width ratio versus longitudinal/transverse sensitivity is conducted (Figure 4a). “b” denotes the length of the sensor and “a” denotes the width of the sensor. As the value of b/a gradually increases, the ratio of the longitudinal sensitivity of the sensor to the transverse sensitivity first increases and then decreases, reaching a maximum at b/a equal to 4.5. The sensitivity in both directions at b/a equal to 4.5 is shown in Figure 4b. The sensitivity in the X-transverse direction is close to zero and the sensitivity in the X-longitudinal direction is much greater than that in the X-transverse direction, so it can be said that unidirectionally sensitive stress sensing is achieved when b/a is 4.5.

### 2.2. Wheatstone Bridge

For easier access to the sensor stress, L-shaped resistors are placed into a Wheatstone bridge. Thus, the measurement of the stress magnitude can be realized by obtaining the voltage output through a simple calibration of the stress versus the output voltage.

Firstly, to ensure that the output of the Wheatstone bridge is zero in the unstressed state, the four resistors on the bridge should be kept equal. Then, to ensure that the bridge has an output in only one axis, the four resistors should be maintained with the same resistance change when stressed in the unresponsive axis of the bridge, making the bridge output still zero. Finally, the voltage output of the bridge can be maximized by placing two L-shaped resistors in the front and back halves of each of the two bridge paths.

**Figure 5:**
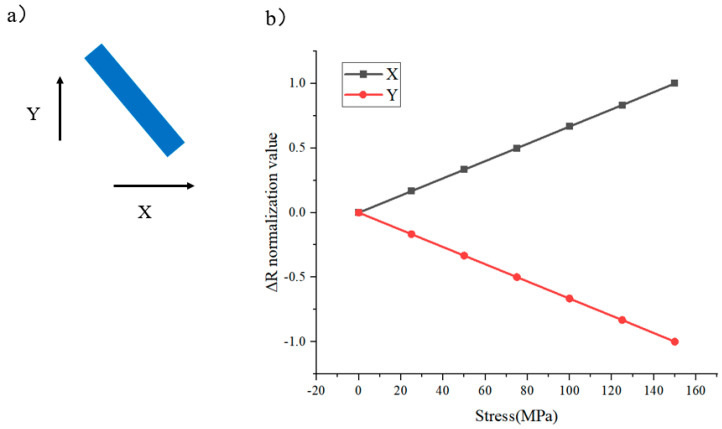
(**a**) 45° resistor and (**b**) its sensitivity in different axes.

Therefore, to compensate for the resistance change of the L-shaped sensor in the non-sensitive axial direction, a 45° bar resistor is introduced here, called the 45°-resistor. This is obtained by rotating the X-bar or Y-bar by 45° and shows the same sensitivity on both axes (Figure 5b).

**Figure 6:**
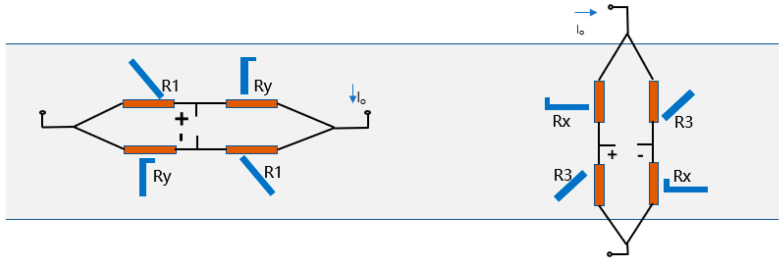
The Wheatstone bridge sensitive to different axes.

Figure 6 shows two bridges that can be read separately to show the magnitude of stresses in different directions. The Wheatstone bridge consists of a first 45°-resistor R1, a first L-shaped resistor Rx/Ry, a second 45°-resistor R1, and a second L-shaped resistor Rx/Ry connected in the first and last orders via a transmission interface. In order to ensure that the voltage output of the bridge is zero in the unstressed state, the R1 resistance should remain equal to the Rx/Ry resistance. Then, in order for the bridge to maintain zero voltage output when stressed in the non-response axis, it is necessary for the four resistors to maintain the same sensitivity in the non-response direction. Therefore, the value of b/a needs to be adjusted again so that Rx/Ry exhibits the same sensitivity in the non-response direction as R1.

The detection of bi-axis stresses within the micro-zone can be achieved by using a combination of X-axis-sensitive integrated stress sensors and Y-axis-sensitive integrated stress sensors in the micro-zone.

**Figure 7:**
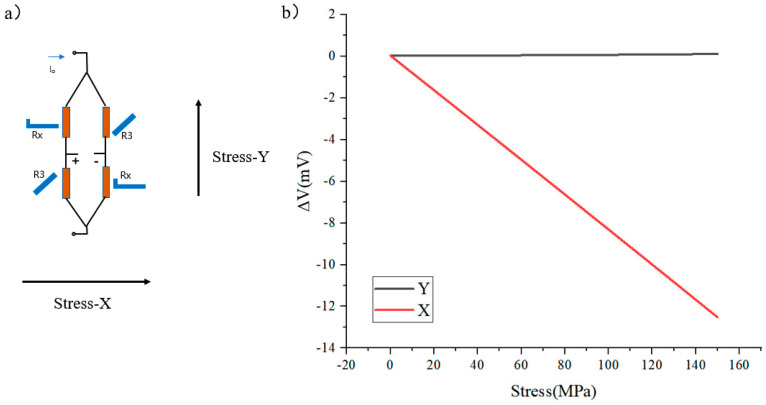
(**a**) X-bridge and (**b**) its simulation voltage output under load.

Theoretically, the response results of a uniaxially sensitive Wheatstone bridge at different stresses are shown in Figure 7. Excited by a voltage of 1 V, this X-bridge maintains an unchanged voltage output when stressed in the y-direction and exhibits a sensitivity of 0.087 mV/V·MPa when stressed in the x-direction. As envisaged, a Wheatstone bridge responding only in a single axis is successfully designed.

**Figure 8:**
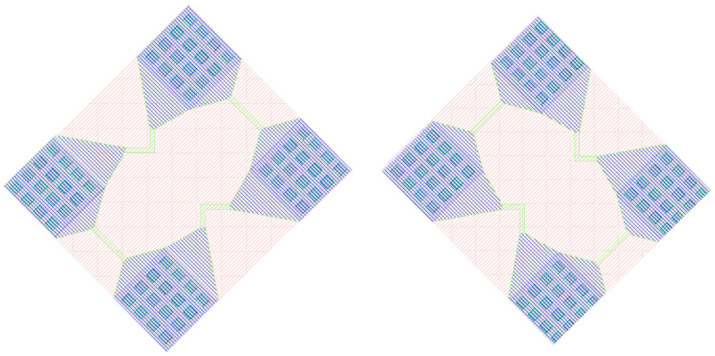
The layout of the one-axis-sensitive Wheatstone bridge.

The layout of the X-bridge and Y-bridge designed in this paper is shown in Figure 8.

### 2.3. Fabrication Process

**Figure 9:**
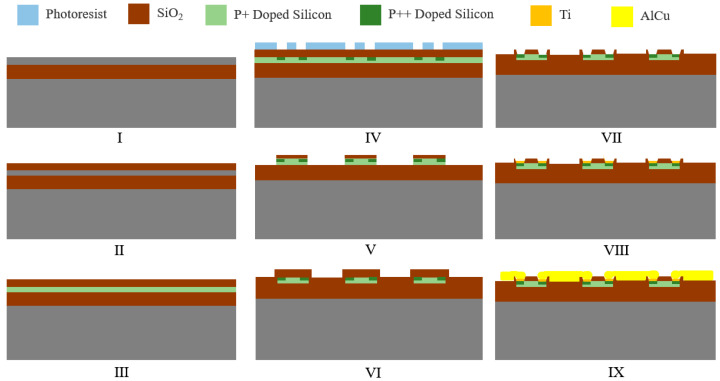
Fabrication process of the sensor.

The fabrication process of the sensor is shown in Figure 9. First, a 725 μm thick SOI wafer is provided with a Si crystal orientation of (110). Second, a 20–30 nm silica layer is grown on the SOI wafer by thermal oxidation. It is then P+-doped using BF_2_^+^ ion implantation (dosage is 2 × 10^14^ and energy is 40 KeV) and rapid thermal annealing at 1080 °C for 30 s. Then, a 2 µm photoresist is spin-coated followed by reactive ion etching and then P++ doping (dosage is 2 × 10^15^ and energy is 25 KeV). The next step is silicon etching. And a layer of 0.2 μm SiO_2_ is deposited on top of the multilayer stack by plasma-enhanced chemical vapor deposition (PECVD). It is then P++-doped by BF_2_^+^ ion implantation (dosage is 10^15^ and energy is 15 KeV) and rapidly thermally annealed at 1080 °C for 10 s. Finally, Ti is deposited and patterned. AlCu is deposited by physical vapor deposition (PVD) and patterned.

### 2.4. Characterization

**Figure 10:**
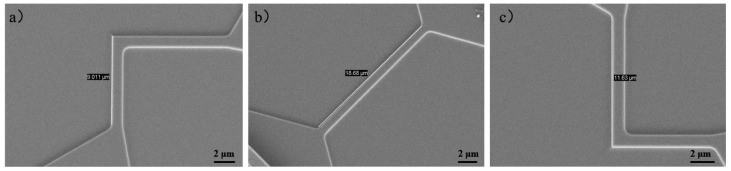
SEM images of resistors.

The sensor seen under a scanning electron microscope (SEM) is shown in Figure 10. X/Y sensors and the 45° resistor are well made.

**Figure 11:**
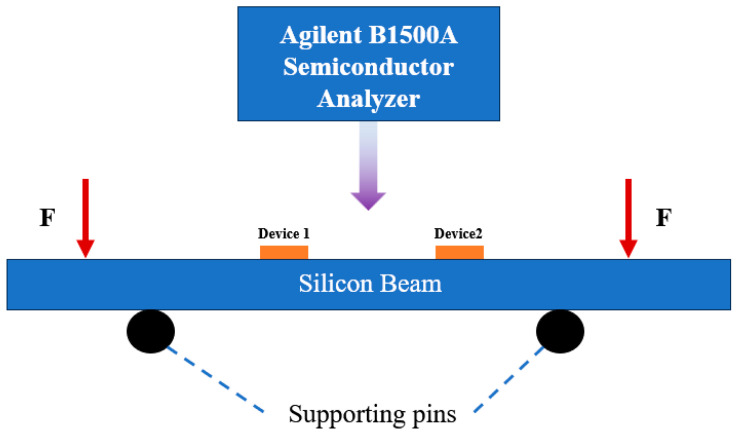
The testing system.

As shown in Figure 11 in this paper, the four-point bending (4PB) method is used to test the stress of the material [35], and the resistance change of the device is tested by an Agilent B1500A semiconductor analyzer (Agilent Technologies, Santa Rosa, CA, USA).

In resistance measurement, the silicon beam is first supported in the middle of the 4PB fixture, and the stress F is applied by the motor control and force sensor, ranging from 0 N to 3 N. The probe is then brought into contact with the resistive gold pads by adjusting the knob, and the force applied by the probe is about 0.02 N. Finally, the signal from the probe is connected by a cable to the semiconductor analyzer for processing.

In the four-point bending test, the distance of the upper support is taken to be 8 cm, and the distance of the lower support is taken to be 5 cm for the present experiments. Due to the relatively long distance between the loading point and the support point, the stress distribution of the beam in the pure bending section is more uniform, showing a more ideal bending state.

## 3. Results

**Figure 12:**
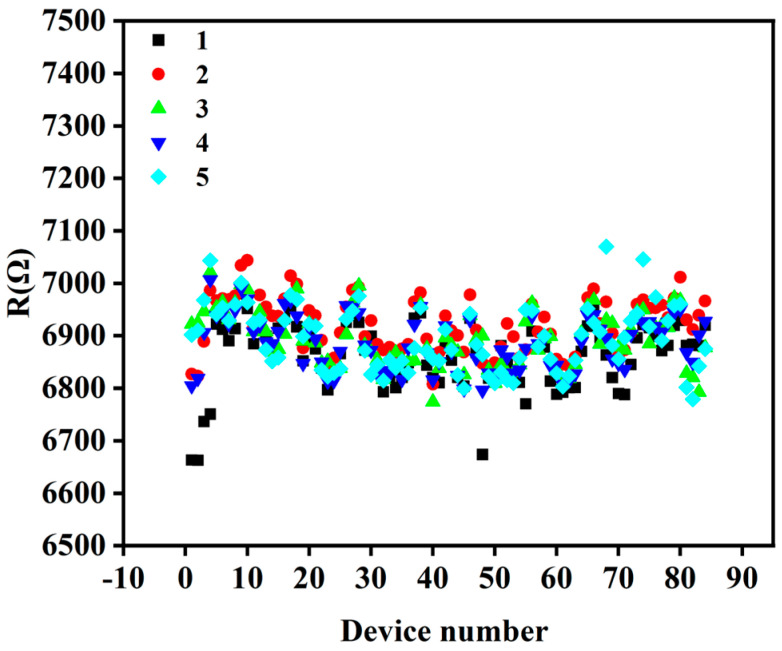
The resistance test values of L-shaped resistors.

Figure 12 shows the resistance test values of L-shaped resistors with an average value of 6891.1 Ω. The horizontal coordinate represents the serial number of the devices tested, and a total of 90 chips from the wafer are tested and five L-shaped resistors are selected for each chip. The resistance values of the resistors at five locations on each device are tested separately.

**Figure 13:**
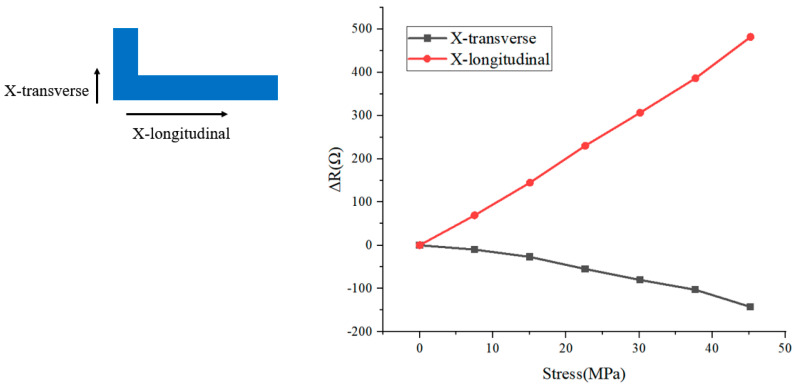
L-shaped resistor’s change in resistance under load.

When the L-shaped resistor is subjected to the same stress in each of the two axes, its resistance change can be measured as shown in Figure 13. It can be seen that there is more than a threefold difference between the sensitivity of the X-longitudinal and X-transverse directions.

**Figure 14:**
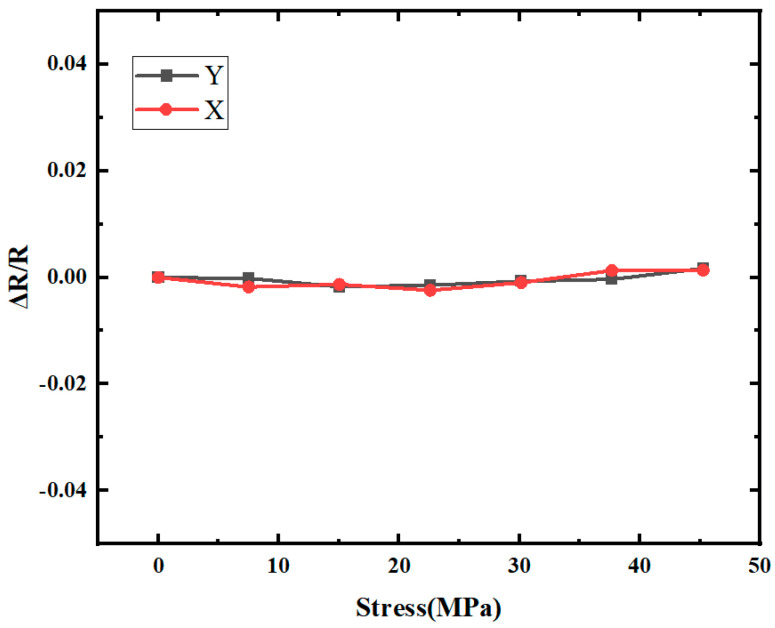
Rate of change in 45°-resistor under load.

Figure 14 shows the test values of the rate of change in resistance for the 45°-resistor. When the 45°-resistor is subjected to the same stress in the X and Y directions, respectively, the rate of change in its resistance is so small that it can be ignored. When the resistance change value of the 45°-resistor under load is very small, the length–width ratio of the L-sensor can be scaled again so that the sensitivity in the sensitive axis can be maximized.

**Figure 15:**
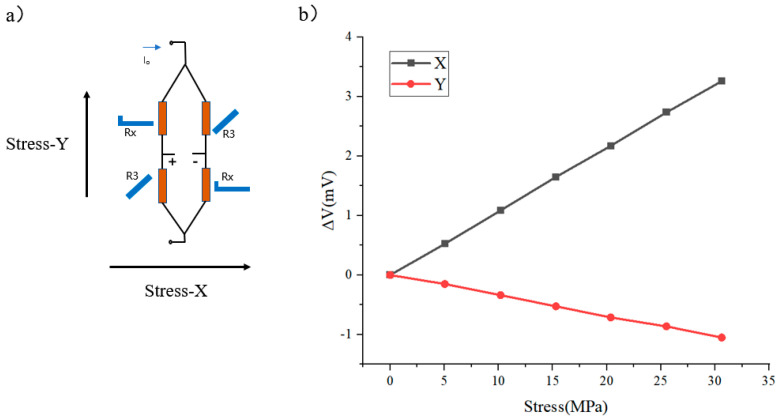
(**a**) X-bridge and (**b**) its voltage output under load.

Figure 15 shows the voltage change when the X-bridge is stressed in two directions. It can be seen that the difference in the sensitivity of the X-bridge in two directions is about three times the sensitivity in the x-direction than in the y-direction; moreover, the X-bridge exhibits good linearity in both stress directions.

The X-bridge still responds in the non-sensitive direction, which is different from the previous design result. Based on this result, the value of b/a can be adjusted again to achieve the result that the X-bridge maintains zero output when stressed in the y-direction. Excited by a voltage of 1 V, this X-bridge exhibits a sensitivity of 0.1 mV/V·MPa when stressed in the x-direction.

**Figure 16:**
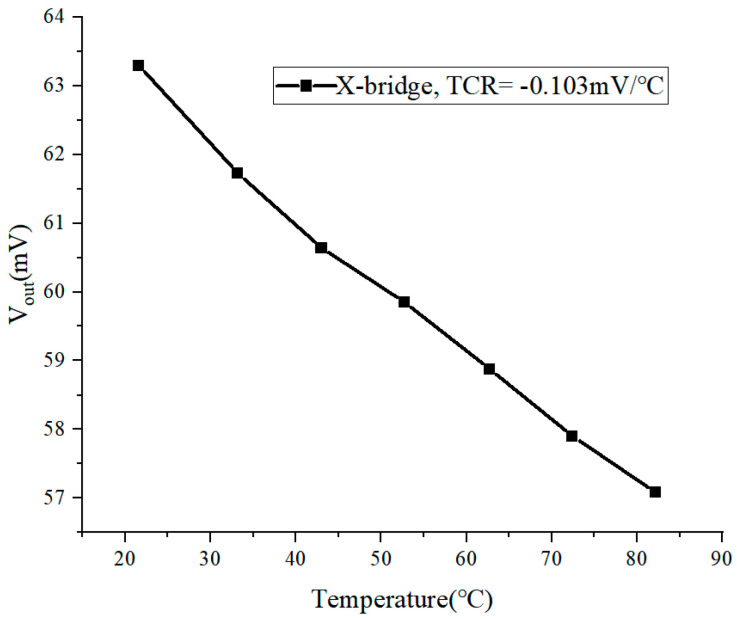
The TCR of the X-bridge.

The temperature coefficient of resistance (TCR) of the X-bridge is −0.103 mV/°C as shown in Figure 16. This may be due to the fact that the ion doping concentrations of the four resistors on the bridge are not guaranteed to be identical during the fabrication process; furthermore, the results do not set aside the effect of thermal stress.

## 4. Discussion

Although the X-bridge shows good sensitivity in sensing stress in the x-direction, its output is not zero in the non-sensitive direction. This may be due to errors in the preparation process: when photolithography is aligned, the crystal directions are notch-aligned (usually exhibits about 5° of deviation), which results in an error in the confirmation of crystal directions, making the experimental results different from the simulation results. The resistors are not perfectly aligned in two mutually perpendicular directions, so the two directions are not completely canceled. The result obtained is not the sensitivity in the ideal direction. In order to improve the alignment accuracy with the (110) and (100) directions, the subsequent process will use KOH anisotropic wet etching. The etching method has different etching rates for different crystal orientations of silicon. By using its etched edge as a photolithography mark, lithography alignment accuracy can be improved.

The output is not actually zero in the state of zero stress and there is an offset of approximately 30 mV in the unstressed state. Firstly, this may be because the simulation results are not accurate enough. In the simulation, the sensitive directions of different resistors do not strictly correspond to the crystal direction, so the differences in resistivity between the (100) and (110) crystal directions are ignored. The actual bridges are prepared with L-shaped resistance in the (110) direction and the 45°-resistor in the (100) direction. Secondly, unavoidable errors in the preparation will also lead to the existence of this compensation; for example, the turn of the L resistor is not at a perfectly ideal right angle, leading to the difference between the actual value and the calculated value. Follow-up work can refine the simulation model, make improvements to the manufacturing process, and fine tune the layout.

The following table (Table 2) briefly compares the results of the work in this paper with those of other findings. It can be seen that this work innovatively achieves uniaxial sensitivity and shows a good sensitivity of 0.16%/MPa.

## 5. Conclusions

In conclusion, the research presented in this paper has successfully developed a real-time uniaxially sensitive stress sensor that addresses the pressing integration challenges and complex signal decoding issues faced by current stress sensors in microsystems. The proposed sensor leverages the simplicity of bar resistor combinations, capitalizing on their difference in sensitivity across various axes, to achieve accurate stress measurement. By employing a Wheatstone bridge circuit, the sensor simplifies the calibration process, correlating stress to output voltage with ease.

The structural and procedural simplicity of the design, coupled with its improved temperature stability and reduced interface requirements, positions this sensor as an ideal solution for health monitoring applications in multi-chip microsystem chips. This innovation represents a significant advancement in the field of microsystem stress sensing, offering a practical and efficient approach to stress detection that is both reliable and cost-effective. As a result, this research contributes to the ongoing development of more robust and intelligent microsystems, paving the way for enhanced performance and longevity in various applications, from industrial to biomedical environments.

## Figures and Tables

**Table 1:** Material parameters used in the simulation.

Parameter Name	Si	SiO_2_
Density (g/cm^3^)	2.23	2.20
Young modulus (GPa)	170	75
Poisson’s ratio	0.28	0.23
Relative dielectric constant	11.7	3.9
Doping concentration (/cm^3^)	10^19^	/

**Table 2:** Comparison of existing piezoresistive stress sensors.

Work	Uniaxial Sensitivity	Piezo-Resistor Sensitivity (%/MPa)	Piezo-Resistor TCR(%/°C)
Gharib et al. [18]	NO	0.0125	0.129
Wang et al. [20]	NO	0.025	0.015
Kayed et al. [36]	NO	0.02	0.202
This work	YES	0.16	0.064

## Data Availability

The original contributions presented in this study are included in the article, and further inquiries can be directed to the corresponding authors.
